# Effects of prebiotics, probiotics and synbiotics on serum creatinine in non-dialysis patients: a meta-analysis of randomized controlled trials

**DOI:** 10.1080/0886022X.2022.2152693

**Published:** 2023-01-13

**Authors:** Fenfen Liu, Yang Liu, Xueai Lv, Hengzhong Lun

**Affiliations:** a Department of Nephrology, The Affiliated Taian City Central Hospital of Qingdao University, Taian, China; b Senior Department of Cardiology, the Sixth Medical Center, Chinese PLA General Hospital, Beijing, China; c Department of Clinical Laboratory, The Affiliated Taian City Central Hospital of Qingdao University, Taian, China

**Keywords:** Chronic kidney disease, meta-analysis, prebiotics, probiotics, synbiotics, creatinine

## Abstract

**Objective:**

Serum creatinine level are influenced by many factors. Although accumulated data suggested that prebiotics, probiotics and synbiotics supplements could affect serum creatinine level, the results remained controversial. The aim of the present paper was to evaluate the effects of prebiotics, probiotics and synbiotics on serum creatinine in non-dialysis patients.

**Methods:**

PubMed, EMBASE (Excerpta Medica Database) and the Cochrane Library databases were searched for eligible randomized, controlled trials (RCTs) which were limited to English language studies until 30 September 2022. A random-effects model was performed to analyze the impact of pooled trials.

**Result:**

Twelve randomized, controlled trial studies were included in the meta-analysis. Prebiotics, probiotics or synbiotics supplementation did not significantly decrease the serum creatinine levels in non-dialysis patients compared to placebo [standardized mean difference (SMD) = 0.05; 95% confidence interval (CI): (−0.21, 0.31); *p* = 0.72; I^2^ = 61%].

**Conclusion:**

The present meta-analysis indicated that supplementation with prebiotics, probiotics and synbiotics could not act as promising adjuvant therapies to decrease the serum creatinine levels in non-dialysis patients.

## 1. Introduction

Creatinine, an efficient biomarker of kidney function, has been widely used clinically for the diagnosis of chronic kidney disease (CKD) and the evaluation of kidney-related drugs. Creatinine is also useful in the diagnosis and functional evaluation of other systemic diseases, including cancer, liver disease, estimation of muscle mass and biomarker of psychiatric disorders [1,2]. Recently, probiotics, prebiotics and synbiotics have become the focus of research. Probiotics are generally defined as live microorganisms, which when administered as food components or supplements confer a benefit on the host [3,4]. Prebiotics are indigestible compounds that selectively promote the growth and reproduction of bacteria such as *Bifidobacterium* and *Lactobacillus* and improve the composition and/or function of the intestinal microbiota [5,6]. Synbiotics are a mixture of probiotics and prebiotics. Many studies have reported the effects of prebiotics, probiotics and synbiotics on renal profile among CKD [7–9]. In addition, the effects of prebiotics, probiotics and synbiotics on creatinine have recently been studied in other diseases. Unfortunately, the results in these studies remain contrary, and in addition, the quality of many of these studies and their evidence bases are debatable [10,11]. Considering that the decrease of serum creatinine could not be identified whether it was due to dialysis process itself or due to the effect of prebiotics, probiotics or synbiotics, we therefore conducted the current meta-analysis of randomized, controlled trials to evaluate the impact of prebiotics, probiotics and synbiotics on serum creatinine levels in non-dialysis patients. Our study will help to clarify whether the use of probiotics, prebiotics and synbiotics can be a promising adjunctive therapy for reducing serum creatinine levels in non-dialysis patients when creatinine is used as biomarkers and other clinical applications.

## 2. Methods

### 2.1. Registration and protocol

This meta-analysis was conducted following the Preferred Reporting Items for Systematic Reviews and Meta-Analyses (PRISMA) Statement [12]. The protocol of this study was registered in the PROSPERO (International prospective register of systematic reviews with the registration number CRD42017064171).

### 2.2. Data Source and search strategy

Two authors (F.F.L., H.Z.L.) independently searched PubMed, EMBASE and the Cochrane Library Central Register of Controlled Trials databases. The online databases were searched until 30 September 2022 and the search was limited to English language studies. The search strategies are provided in Supplementary Table 1. In addition, references of selected studies and relevant review articles were searched for additional eligible articles.

### 2.3. Selection criteria

We included trials with the following criteria: (1) Type of study: randomized controlled trial on humans; (2) Participants: restricted to patients aged >18 years; (3) Intervention: patients were treated with prebiotics, probiotics, or synbiotics for at least 3 weeks; (4) Outcome: assessed blood parameters of creatinine. We excluded studies with the following characteristics: (1) the studies had no clear inclusion or exclusion criteria; (2) Patients undergoing hemodialysis or peritoneal dialysis; (3) uncontrolled studies.

### 2.4. Data Extraction

Each study was independently reviewed by two independent researchers. Firstly, we screened the titles and abstracts of all potentially relevant studies. Then the full text of the studies was examined in terms of the exclusion criteria. The final included articles were determined by discussion between the two reviewers, and any disagreements were reconciled by reaching a consensus. The following data was extracted from each selected study: study name, authors, year of publication, type of study design, intervention measures, trial duration, total number of participants, age, sex, change in serum creatinine and related diseases. The change in serum creatinine levels before and after interventions was analyzed with the summary statistics of the relevant studies translated into mean differences and standard deviations (MD ± SD) or post-treatment mean and SD (means ± SD). For an included randomized crossover controlled trial, the final evaluation data were extracted.

### 2.5. Statistical analysis

Data was analyzed using RevMan software (Cochrane Review Manager, version 5.3) to evaluate the effects of prebiotics, probiotics and synbiotics on serum creatinine in non-dialysis patients. The outcomes were presented as SMD with 95% CI. Considering the additional uncertainty associated with the effect of treatment of different interventions, a random-effects model (DerSimonian–Laird method) was used to calculate the pooled estimates of the mean differences in serum creatinine levels. A P value <0.05 was considered statistically significant. Inter-study heterogeneity was assessed by the Cochran Q statistics and quantified by the I^2^ statistics. The magnitude of heterogeneity was classified as as I^2^ ≤ 40%%: low, 40% < I^2^ ≤ 70%%: moderate and I^2^ > 70% [13]: high. Risks of bias in RCTs were assessed using the Cochrane Risk of Bias 2 assessment tool which contain the following items: randomization process; deviations from intended interventions; missing outcome data; measurement of the outcome; selection of the reported result and overall [14]. Sensitivity analysis was used to test the stability of the results, and it was performed by omitting the trials one by one to identify which RCTs caused the heterogeneity and how each contributed to the overall result. Funnel plots were constructed to assess the publication bias. GRADE (Grading of Recommendations Assessment, Development and Evaluation) approach was used to assess the study quality.

## 3. Results

### 3.1. Characteristics of included studies

**Figure 1.**
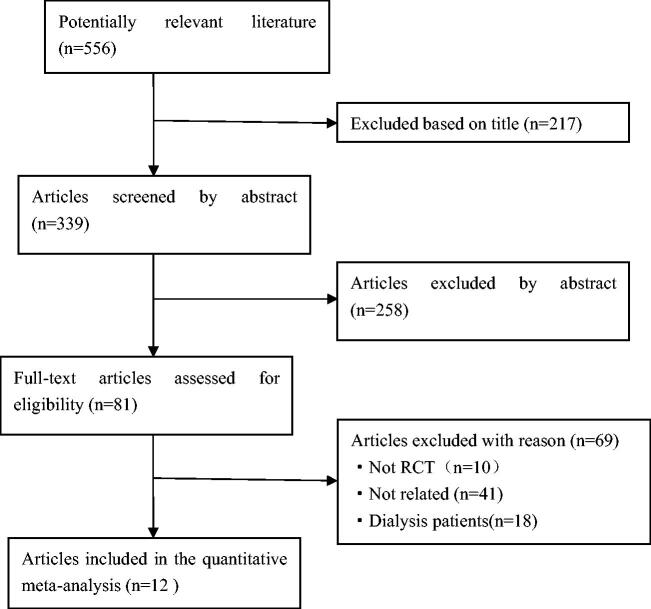
Study flow diagram.

After searching PubMed, EMBASE, the Cochrane Library and related grey literatures, 556 articles were obtained. Of these, 217 were excluded based on the title (including overlapping articles and reviews), 258 were excluded based on the abstract, leaving 81 articles for full text review. By reviewing the full text, 69 papers were discarded based on the exclusion criteria. Finally, twelve eligible studies were incorporated into the meta-analysis. Figure 1 showed a flow diagram of the search methodology. All twelve included studies were randomized controlled trials：seven were about kidney disease, two were about diabetes, one was about hypertension, one was about Japanese cedar pollinosis patients, and one was performed in neurocritical care patients. Among all types of interventional trials, four used probiotics, three used prebiotic and five used synbiotics. Trial characteristics are detailed in Table 1.

**Table 1.** Characteristics of Included Studies.

Study, Year	Type of Study	Total sample (case: control)	Sex (M/F)	Mean Age (years)	Intervention	Duration	serum creatinine level after treatment (mg/dL)	related diseases
Firouzi et al. (2015) [15]	Randomized, Controlled parallel trial	136 (68/68)	71/65	53	Probiotic supplement 6 × 10^10^colony-forming unit (CFU)/d	12 weeks	Intervention Group: 0.82 ± 0.22 Control Group: 0.85 ± 0.21	Diabetes
Dehghani et al. (2016) [16]	Randomized, Controlled parallel trial	66 (31/35)	50/16	61	Synbiotics supplement, 1000 mg/d	6 weeks	Intervention Group: 1.90 ± 0.70 Control Group: 2.18 ± 1.14	Kidney disease
Rossi et al. (2016) [17]	Randomized, Controlled crossover trial	31 31/31)	21/10	69	Synbiotics supplement The first 3 weeks, 7.5 g placebo/prebiotic powder and one placebo/probiotic capsule containing 4.5 × 10^10^ CFU. After the dose escalation, 15 g and 9 × 10^10^CFU/d for 6 weeks	9 weeks	Intervention Group: 2.61 ± 0.85 Control Group: 2.64 ± 0.84	Kidney disease
Abbasi et al. (2017) [18]	Randomized, controlled parallel trial	40 (20/20)	NA	55	Probiotic supplement 4 × 10^9^ CFU/d	8 weeks	Intervention Group: 0.83 ± 0.16 Control Group: 1.00 ± 0.14	Kidney disease
Ebrahimi et al. (2017) [19]	Randomized, controlled parallel trial	70 (35/35)	42/28	59	Synbiotics supplement 500 mg/d	9 weeks	Intervention Group: 1.05 ± 0.26 Control Group: 1.03 ± 0.24	Diabetes
Harata et al. (2017) [20]	Randomized, controlled parallel trial	25 (14/11)	9/16	37	Probiotic supplement	10 weeks	Intervention Group (SE): 0.7 ± 0.2 Control Group (SE): 0.7 ± 0.2	Cedar pollinosis patients
Tuncay et al. (2018) [21]	Randomized, controlled parallel trial	46 (23/23)	23/23	71.8	Prebiotic supplemen	3 weeks	Intervention Group: 1.23 ± 0.84 Control Group: 0.77 ± 0.37	Neurocritical care patients
Ramos et al. (2019) [22]	Randomized, controlled parallel trial	46 (23/23)	27/19	57	Prebiotic supplement 12g/d	3 months	Change of intervention Group (Range): (−0.2 to 0.3) Change of control Group (Range): 0.0 (−0.1 to 0.3)	Kidney disease
Romão et al. (2020) [23]	Randomized, controlled parallel trial	36 (19/17)	0/36	43	Probiotic supplement 4 × 10^9^ CFU/d	8 weeks	Intervention Group (95% CI): 0.65 (0.56–0.73) Control Group (95% CI): 0.58 (0.52–0.63)	Hypertension
Cosola et al. (2021) [24]	Randomized, controlled parallel trial	*n* = 23 (13/10)	14/9	51	Synbiotics supplement probiotics 9.6 × 10^9^ CFU/d prebiotics 10 g/d	2 months	intervention Group (SE): 2.3 ± 0.2 control Group (SE): 3.1 ± 0.4	Kidney disease
Armani et al. (2021) [25]	Randomized, controlled parallel trial	46 (23/23)	24/22	57.6	Prebiotic supplement 12g/d	3 months	Intervention Group (Range): 2.96 (2.00–3.53) Control Group (Range): 2.83 (2.34–3.75)	Kidney disease
McFarlane et al. (2021) [26]	Randomized, controlled parallel trial	56 (28/28)	NA	70	Synbiotics supplement The first 2 weeks, prebiotic 10 g /d, probiotic 4.5 × 10^11^ CFU/d. Prebioti 20 g /d, probiotic 4.5 × 10^11^ CFU/d for 50 weeks.	52 weeks.	Change of intervention Group (95%CI): 0.24 (0.03–0.44) Change of control Group (95%CI): −0.11 (−0.25–0.02)	Kidney disease

Data are presented as means ± SD, means ± SE, means (Range), means (95% CI); CI: confidence interval; M: male; F: female; NA: not available.

### 3.2. Main outcomes

We conducted meta-analysis with twelve randomized, controlled trials. Subgroup analysis was performed by the types of interventions, treatment duration and the type of clinical trial studies. Moreover, we assessed the effects of biotic (prebiotic, probiotic, synbiotics), synbiotics supplements and biotic treatment duration longer than 2 months on serum creatinine in non-dialysis kidney disease patients.

#### 3.2.1. Effects of prebiotics, probiotics and synbiotics on serum creatinine in non-dialysis patients

Twelve eligible studies including 652 participants were presented the pooled effect of prebiotic, probiotic, and synbiotic supplementation on the serum creatinine levels in non-dialysis patients.

**Figure 2.**
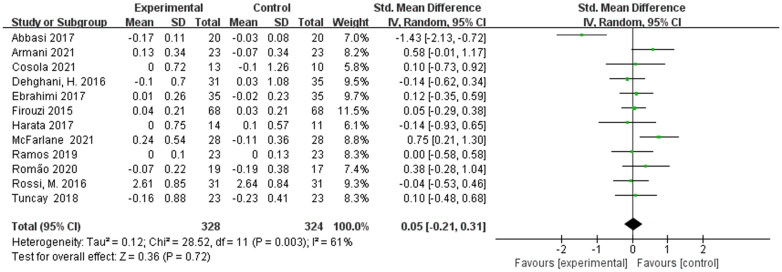
Meta-analysis for prebiotics, probiotics and synbiotics on serum creatinine in non-dialysis patients.

Meta-analysis showed that the post-treatment serum creatinine levels were not decreased significantly in non-dialysis patients [SMD = 0.05; 95% CI: (−0.21, 0.31); *p* = 0.72; I^2^ = 61%]. The heterogeneity was moderate and sensitivity analysis revealed that omitting a single study had no influence on the total result not be adjusted by sensitivity analysis. The pooled effect was shown in Figure 2.

#### 3.2.2. Effects of different types of interventions on serum creatinine in non-dialysis patients

**Figure 3.**
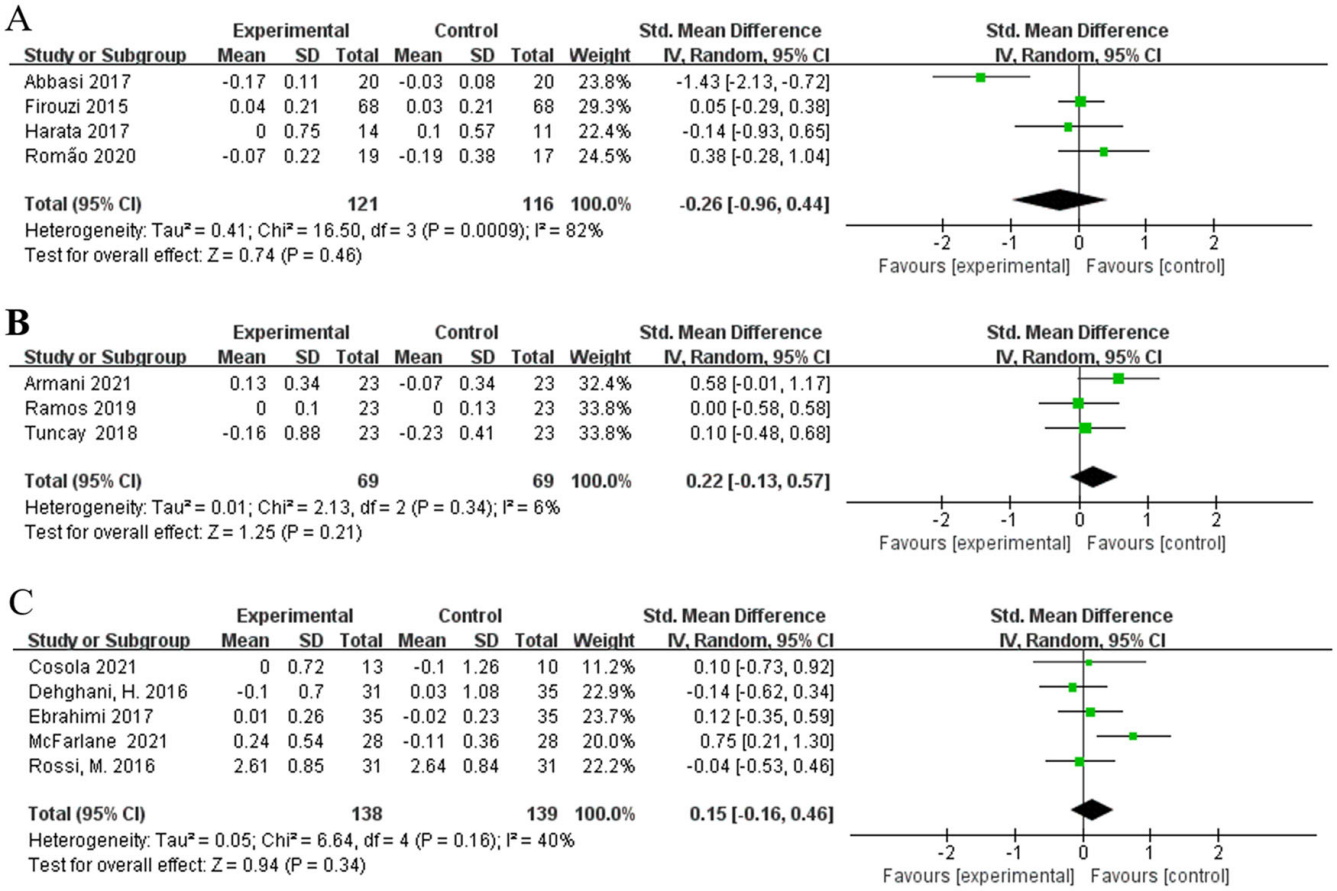
Effects of different types of interventions on serum creatinine. (A) Effects of probiotics on serum creatinine. (B) Effects of prebiotics on serum creatinine. (C) Effects of synbiotics on serum creatinine.

Subgroup analysis identified the different type of interventions as one source of intergroup heterogeneity. Four studies [15,18,20,23] were involved in the assessment of effects of probiotics on serum creatinine in non-dialysis patients and no statistically significant difference was found [SMD = −0.26; 95%CI: (−0.96, 0.44); *p* = 0.46] with a high heterogeneity (I^2^ = 82%) (Figure 3(A)). Both prebiotics subgroup [21,22,25], [SMD = 0.22; 95%CI: (−0.13, 0.57); *p* = 0.21; I^2^ = 6%] and synbiotics subgroup [16,17,19,24,26], [SMD = 0.15; 95%CI: (−0.16, 0.46); *p* = 0.34; I^2^ = 40%] did not show a significant decrease in serum creatinine level (Figure 3(B,C)). The heterogeneity of the SMDs was considered low in prebiotics subgroup and synbiotics subgroup.

#### 3.2.3. Effects of different treatment duration on serum creatinine in non-dialysis patients

**Figure 4.**
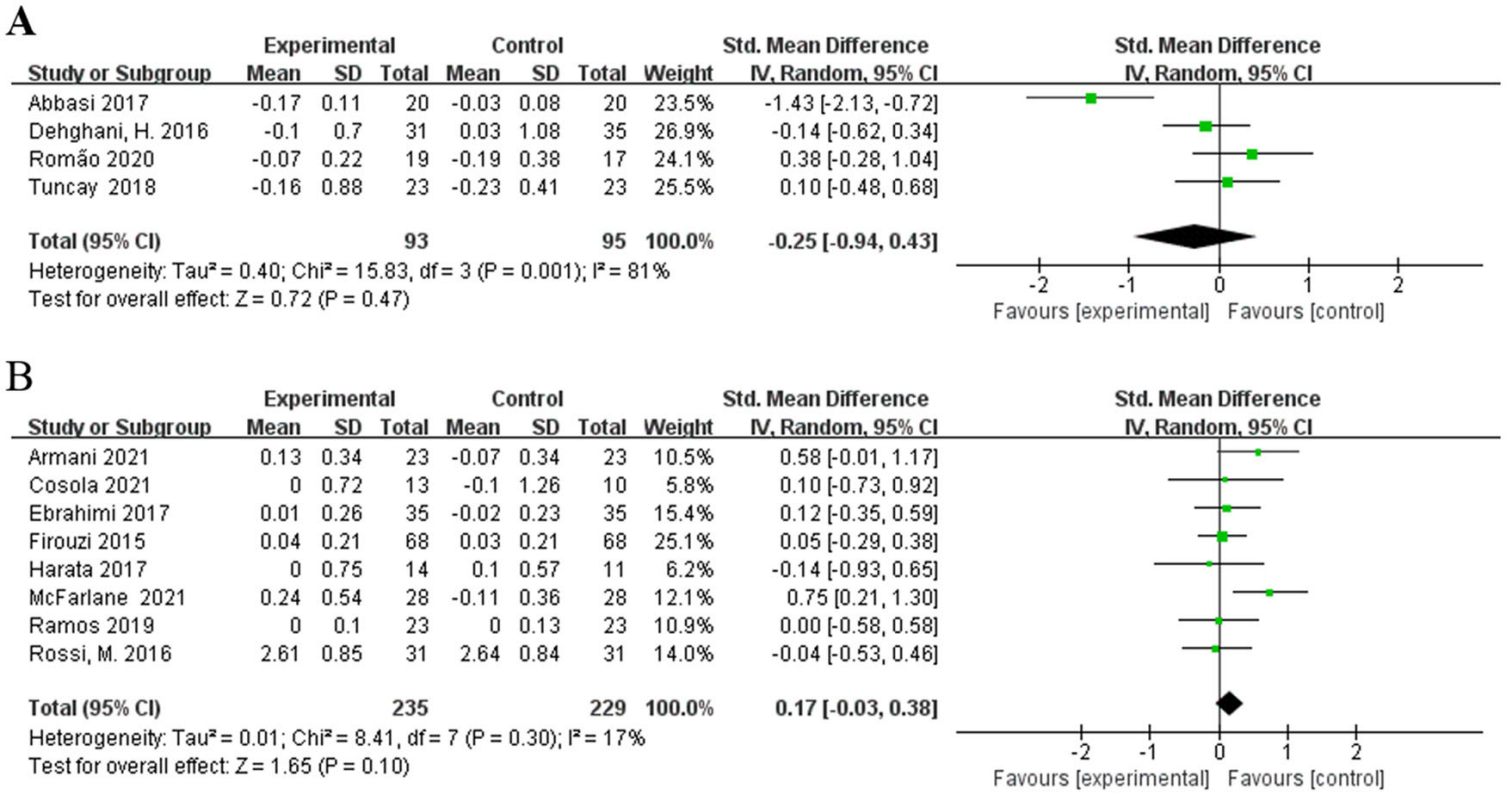
Effects of different treatment duration on serum creatinine. (A) Effects of treatment duration 2 months or less on serum creatinine. (B) Effects of treatment duration longer than 2 months on serum creatinine.

Considering the effect of treatment duration on outcome, subgroup analysis was conducted according to treatment duration as another source of intergroup heterogeneity. Four studies [16,18,21,23] involving 188 patients were included in the meta-analysis for treatment durations 2 months or less. Pooled meta-analysis showed that no evidence indicated a significant difference between intervention and control group [SMD = −0.25; 95%CI: (−0.94, 0.43); *p* = 0.47; I^2^ = 81%] (Figure 4(A)). The heterogeneity of the SMDs was considered high. Eight RCTs [15,17,19,20,22,24–26] with biotic intervention longer than 2 months also showed no significant difference in their meta-analysis [SMD = 0.17; 95%CI: (−0.03, 0.38); *p* = 0.10; I^2^ = 17%] (Figure 4(B)). Heterogeneity was removed with a duration more than 2 months.

#### 3.2.4. Effects of different type of clinical trial studies on serum creatinine in non-dialysis patients

**Figure 5.**
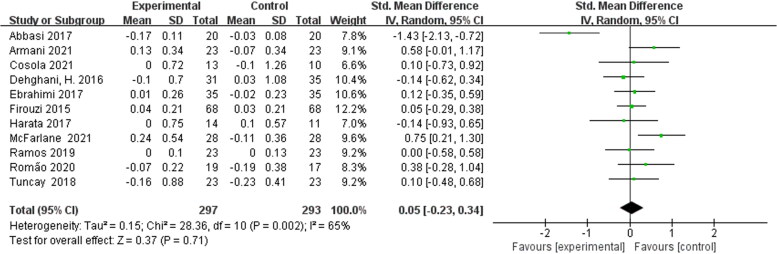
Meta-analysis of randomized controlled parallel trials for prebiotics, probiotics and synbiotics on serum creatinine in non-dialysis patients.

Of the twelve included studies, eleven studies were randomized controlled parallel trials and one study [17] was randomized controlled crossover trials. We performed a meta-analysis of these eleven randomized controlled parallel trials. Pooled meta-analysis showed that biotic treatment did not significantly reduce serum creatinine levels in non-dialysis patients [SMD = 0.05; 95% CI: (−0.23, 0.34); *p* = 0.71; I^2^ = 65%] (Figure 5). Moderate heterogeneity occurs among these studies.

#### 3.2.5. Effects of prebiotics, probiotics and synbiotics on serum creatinine in non-dialysis kidney disease patients

**Figure 6.**
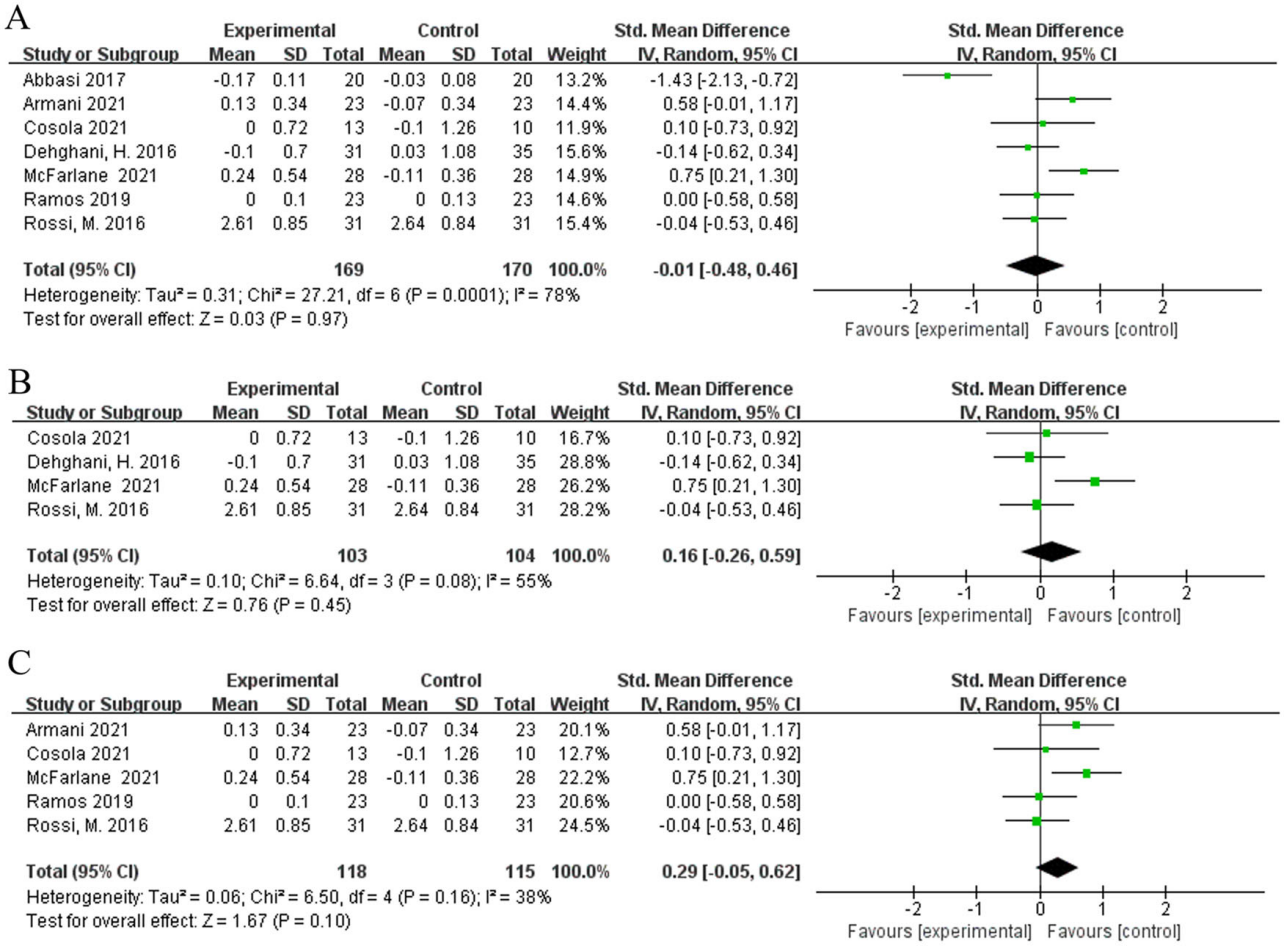
Meta-analysis for prebiotics, probiotics and synbiotics on serum creatinine in non-dialysis kidney disease patients. (A) Effects of of prebiotics, probiotics and synbiotics on serum creatinine in kidney disease patients. (B) Effects of of synbiotics on serum creatinine in kidney disease patients. (C) Effects of treatment duration longer than 2 months on serum creatinine in kidney disease patients.

Seven studies [16–18,22,24–26] evaluated the effects of biotic interventions on serum creatinine in non-dialysis kidney disease patients. Pooled meta-analysis showed that no evidence indicated a significant difference between biotic and placebo [SMD = −0.01; 95%CI: (−0.48, 0.46); *p* = 0.97; I^2^ = 78%] (Figure 6(A)). Subgroup [16,17,24,26] analysis showed that supplementation of synbiotics did not decrease the serum creatinine levels in non-dialysis kidney disease patients compared to the placebo [SMD = 0.16; 95%CI: (−0.26, 0.59); *p* = 0.45; I^2^ = 55%] (Figure 6(B)). We did not perform a subgroup analysis for probiotics and prebiotics because of the involvement of small number of studies. volvement of small number of studies. In addition, we combined five studies [17,22,24–26] in patients with non-dialysis kidney disease intervented duration for longer than 2 months and found no significant difference in their meta-analysis [SMD = 0.29; 95%CI: (−0.05, 0.62); *p* = 0.10; I^2^ = 38%] (Figure 6(C)).

### 3.3. Publication bias

**Figure 7.**
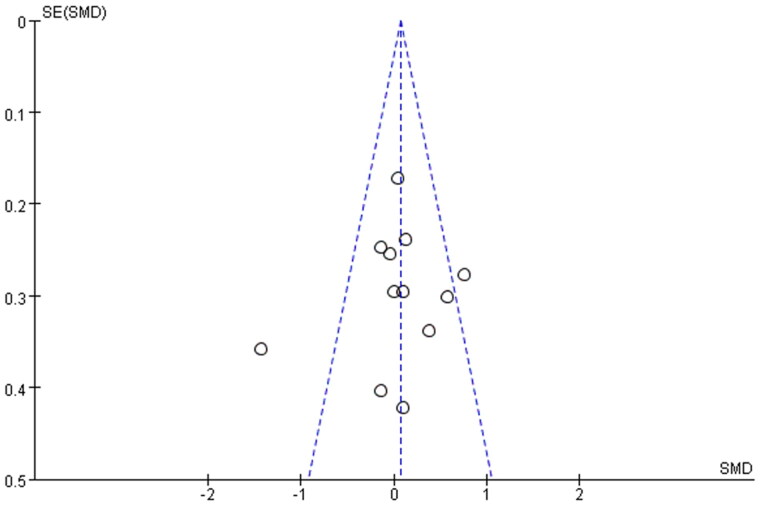
Funnel plots of the meta-analysis for prebiotics, probiotics and synbiotics on serum creatinine levels.

Funnel plots were constructed to assess the publication bias and identify sources of heterogeneity (Figure 7). The obtained funnel plots presented no proof of obvious publication bias for the included studies. Two studies [18,26] fall outside the funnel plot, suggesting that these two studies are the main source of heterogeneity.

### 3.4. Risk of bias assessment

**Figure 8.**
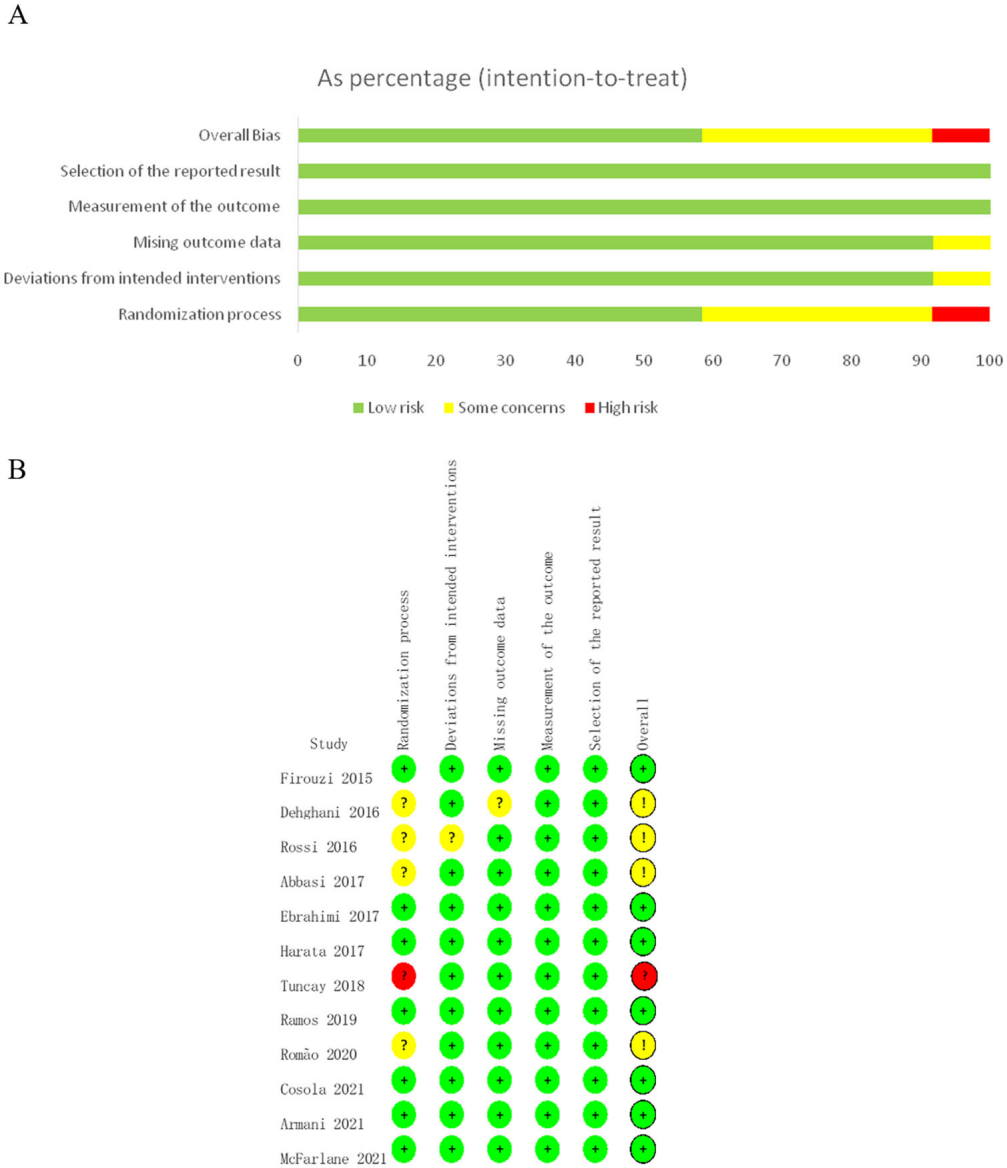
Risk of bias assessment for included RCTs. (A) Risk of bias summary. (B) Risk of bias graph. Green indicated low risk of bias, yellow for some concerns of bias and red for high risk of bias.

According to the Cochrane risk-of-bias tool for randomized trials version 2, ‘Selection of the reported result’ and ‘Measurement of outcome bias’ was assessed as low risk in all the included RCTs (Figure 8(A)). Seven studies were considered as ‘low risk bias’, four studies were considered as ‘some concerns of bias’, one study was judged as ‘high risk of bias’ (Figure 8(B)). According to the GRADE quality of evidence evaluation, the effect of probiotics and synbiotics on serum creatinine in non-dialysis patients was evaluated as moderate quality evidence.

## 4. Discussion

The meta-analysis of randomized, placebo-controlled trials demonstrated that supplementation with prebiotics, probiotics and synbiotics cannot reduce the concentrations of serum creatinine in non-dialysis patients. Relevant papers which were conducted in the dialysis patients were excluded because the decline of serum creatinine could not be identified whether it was due to dialysis process itself or due to the effect of prebiotics, probiotics or synbiotics. Moreover, in clinical practice of CKD, serum concentration of creatinine acts as a significant parameter of muscle mass in end-stage renal disease patients undergoing dialysis. The prebiotics, probiotics and synbiotics supplementation in dialysis patients could affect the evaluation of muscle mass.

The impact of probiotics, prebiotics and synbiotics on serum creatinine was highly dependent on the type of probiotic strains and the dosage amount used. The types of interventions of the studies included in this meta-analysis were different. Subgroup analysis did not showe a significant reduction in serum creatinine. The dose of interventions used in the trials also affected the effects of prebiotics, probiotics and synbiotics on serum creatinine in chronic kidney disease patients. A study [27] reported that 16 x 10^9^ CFU dose of probiotics, showed better results compared to 8 x 10^9^ CFU, which showed a significant decrease of almost 11% of the serum urea concentration compared with the baseline values. But the higher dose did not significantly decrease the levels of serum creatinine.

An important purpose of adjuvant therapy with probiotics, prebiotics and synbiotics for patients is to restore the patient’s intestinal flora to a normal state. The restoration of intestinal flora requires a long-term process, so the duration of the intervention can have a significant impact on the treatment outcome. The RCTs included in this study spanned a wide range of treatment durations, from only three weeks of intervention to up to 52 weeks. We performed a subgroup analysis of RCTs with a time point of two months. The results of the combined meta-analysis showed no statistically significant difference in serum creatinine between the treatment and control groups according to the different treatment durations combined, but the subgroups with treatment durations more than two months had lower P values. This suggested that the longer treatment time contributed somewhat to the treatment outcome, but the help is limited.

High heterogeneity was present in some of the meta-analyses conducted in this study. After subgroup analysis based on intervention duration and intervention type, heterogeneity was low in the treatment duration >2 months and in the treatment groups using prebiotics or synbiotics, indicating that intervention duration and interventions generate heterogeneity when meta-analyses are performed. We found that the source of heterogeneity was mainly from the two studies of Abbasi et al. [18] and McFarlane et al. [26]. The heterogeneity was eliminated when these two studies were excluded. In study Abbasi et al. probiotic treatment significantly reduced serum creatinine levels compared with the control group (*p* < 0.01), whereas in the other studies the effect of biotic treatment was not significant, resulting in heterogeneity. The duration of treatment in study McFarlane et al. was 52 weeks, which may account for the heterogeneity in this RCT. In addition, the results of meta-analyses were not changed after sensitivity analysis by removing each study, which indicates that our results are stable and reliable.

Recent studies have suggested that the gut microbiota is one of the pathogenic factors in kidney disease [28]. Vaziri and his colleague demonstrated that uremia profoundly alters the intestinal microbial flora and the relationship between the human microbiome and kidney disease remains bidirectional [29]. Alterations in the composition of microbiome and accumulation of gut derived uremic toxins (such as indoxyl sulfate (IS), *p-*cresyl sulfate (PCS), lipopolysaccharides, amines, ammonia, and trimethylamine oxide) contribute to the systemic inflammation, cardiovascular diseases and numerous other CKD associated complications [30–32]. Probiotics, prebiotics and synbiotics, which are usually considered safe for several diseases, remain crucial for the maintenance of balance of human intestinal microbiota. The supplementation of prebiotics, probiotics or synbiotics could improve the dysbiosis and/or increase the permeability of the intestinal barrier in CKD patients, contributing to the reduction in the levels of uremic toxins. Our meta-analysis indicated that supplementation of prebiotics, probiotics and synbiotics can not significantly decrease the levels of serum creatinine. This result may be for the reasons that creatinine is affected by many factors.

Creatinine is the end product of creatine and creatine phosphate metabolism and the synthesis of this primarily takes place in the liver [33]. Creatine is mainly taken up by the muscle after releasing into the circulation. Two meta-analyses reported that serum creatinine unchanged after fiber or probiotic supplementation in patients with CKD [34,35], which is consistent with our conclusion. In contrast to the findings of our study, meta-analysis reported by Liu, J. et al. found serum creatinine decreased after biotic supplementation in dialysis patients [36], possibly because which was conducted in the dialysis patients. Another recent meta-analysis [37] also reported that probiotics decreased serum creatinine in diabetic kidney disease, probably because this meta-analysis included only studies of diabetic nephropathy and did not exclude dialysis patients. To date, there were very few RCT studies regarding the effect of prebiotics, probiotics and synbiotics on serum creatinine in dialysis patients. Only 12 randomized, controlled trials were involved in our meta-analysis, which might in turn affect the overall results of the meta-analysis. More studies with larger sample groups are necessary for future work.

This study has some limitations. We included a variety of patients who had not undergone dialysis including renal disease, diabetes, hypertension etc in our sudy. Although this allows for a more complete inclusion of the literature it also leads to heterogeneity. Another limitation is that the effect in many occasions was assessed. Although we performed a subgroup analysis there was still a moderate-to-hingh among some studies. In addition, we did not analyze effects of probiotics, prebiotics and synbiotics on serum creatinine in non-dialysis kidney disease patients by CKD stage because of the involvement of small number of studies. The strength of this meta-analysis is that the included studies were all non-dialysis patients, which excludes the effect of dialysis on treatment outcomes. In addition, we analyzed the treatment effects in terms of intervention time, intervention type, trial type, and disease type, all of which yielded the same outcome, reinforcing the point that biotic treatment did not reduce the patients’ creatinine levels.

## 5. Conclusions

In conclusion, prebiotics, probiotics and synbiotics supplementation can not decrease the levels of serum creatinine and these interventions may not act as promising adjuvant therapies in non-dialysis patients. More randomized, controlled studies with larger sample groups are needed to consolidate our conclusions.

## Supplementary Material

Supplemental Material

## Data Availability

The datasets used and/or analyzed during the current study are available from the corresponding author on reasonable request.
