# Supplementary material for: Effects of prebiotics, probiotics and synbiotics on serum creatinine in non-dialysis patients: a meta-analysis of randomized controlled trials
Source: Ren Fail. 2023 Jan 13;45(1):2152693. doi: 10.1080/0886022X.2022.2152693 (PMC9848283; doi:10.1080/0886022X.2022.2152693)
Supplement: Supplemental Material [file IRNF_A_2152693_SM7738.pdf]

Supplementary Table 1. Search strategy in CENTRAL, PubMed and EMBASE databases

**CENTRAL**
#1 MeSH descriptor: [Prebiotics] explode all trees
#2 MeSH descriptor: [Probiotics] explode all trees
#3 MeSH descriptor: [Synbiotics] explode all trees
#4 MeSH descriptor: [Lactobacillus] explode all trees
#5 MeSH descriptor: [Inulin] explode all trees
#6 MeSH descriptor: [Bifidobacterium] explode all trees
#7 (probiotic or probiotics or prebiotics or prebiotic or synbiotics or synbiotics or lactobacillus or lactobacilli or bifidobacterium or bifidobacteria or oligofructose or inulin):ti,ab,kw (Word variations have been searched)
#8 MeSH descriptor: [Creatinine] explode all trees
#9 (creatinine):ti,ab,kw (Word variations have been searched)
#10 #1 OR #2 OR #3 OR #4 OR #5 OR #6 OR #7
#11 #8OR #9
#12 #10 AND #11
**PubMed**
((((((((((((((((((((((((Probiotics[MeSH Terms]) OR (Probiotics[Text Word])) OR (Probiotic[Text Word])) OR (Prebiotics[MeSH Terms])) OR (Prebiotics[Text Word])) OR (Prebiotic[Text Word])) OR (Synbiotics[MeSH Terms])) OR (Synbiotics[Text Word])) OR (Synbiotic[Text Word]))) OR (lactobacillus[MeSH Terms])) OR (Lactobacillus[Text Word])) OR (lactobacilli[Text Word])) OR (Bifidobacterium[MeSH Terms])) OR (Bifidobacterium[Text Word])) OR (bifidobacteria[Text Word])) OR (oligofructose[Text Word])) OR (Inulin[MeSH Terms])) OR (Inulin[Text Word])) AND ((creatinine[MeSH Terms]) OR (creatinine[Text Word]))) AND ((((Randomized Controlled Trial[MeSH Terms]) OR (Randomized Controlled Trial[Text Word])) OR (Randomized[Text Word])) OR (placebo[Text Word]))
**EMBASE**
#1. 'prebiotics' OR 'prebiotic':ab,kw,ti
#2. 'synbiotics' OR 'synbiotic':ab,kw,ti
#3. 'probiotics' OR 'probiotic':ab,kw,ti
#4. 'lactobacillus'/exp OR 'lactobacillus':ab,kw,ti
#5. 'lactobacilli':ab,kw,ti
#6. 'bifidobacterium'/exp OR 'bifidobacterium' OR 'bifidobacteria':ab,kw,ti
#7. 'oligofructose'/exp OR 'oligofructose':ab,kw,ti
#8. 'inulin'/exp OR 'inulin':ab,kw,ti
#9. e'cr atinine'/exp OR creatinine
#10. 'randomized controlled trial':ab,kw,ti
#11. 'randomized controlled trial'/exp
#12. #1 OR #2 OR #3 OR #4 OR #5 OR #6 OR #7 OR #8
#13. #10 OR #11
#14. #9 AND #12 AND #13
